# Preliminary Design Analysis of Membrane Bioreactors Application in Treatment Sequences for Modernization of Wastewater Treatment Plants

**DOI:** 10.3390/membranes12090819

**Published:** 2022-08-23

**Authors:** Nikolay Makisha

**Affiliations:** Research and Education Centre “Water Supply and Wastewater Treatment”, Moscow State University of Civil Engineering, 26, Yaroslaskoye Highway, 129337 Moscow, Russia; makishana@mgsu.ru

**Keywords:** wastewater treatment, membrane bioreactor, modernization, activated sludge reactor, cost analysis

## Abstract

By using modeling with the Capdetworks software package, the study examines the definition of the essential elements of operational expenses at wastewater treatment facilities with a capacity of 1 to 100 thousand cubic meters per day. Four different treatment sequences were examined in the study; the first three revealed a standard setup with an activated sludge reactor and secondary clarifier (operating under various operating conditions), and the fourth scheme combined an activated sludge reactor with a submerged membrane bioreactor for sludge separation. The values of concentrations of key pollutants common for urban wastewater before treatment as well as technological parameters of operation were utilized as initial data for calculations because it was crucial to obtain conclusions that could be applied at real facilities. For each of the four treatment sequences, values for pollutants concentrations in effluent wastewater and hydraulic retention time were obtained and analyzed. The expenses of operating biological treatment facilities and treatment facilities in general, as well as the specific cost of power for treating 1 m^3^ of wastewater, were taken into account. Additionally, the price of purchasing membrane modules, which can be categorized as operational due to their replacement frequency of around every 7 to 10 years, was determined. The study’s findings demonstrated that the use of membrane technologies at the secondary treatment stage might significantly affect the rebuilding of wastewater treatment plants under conditions of increased capacity (flow rate) and constrained area for growth.

## 1. Introduction

In current practice of wastewater system design, it is accepted that the main part of wastewater volume is generated from household activities of the population (both in houses and at workplaces) [1]. The volume of domestic wastewater can be estimated based on the value of the daily rate of drainage (L per capita). While the consumed tap water can be normally measured by metering devices, specific water consumption and wastewater disposal can be approximately assumed to be equal to each other [2]. However, this approach cannot be considered the most precise. Specific water consumption is affected by a variety of factors, including high outdoor air temperature, settlement size, varying standard of living, hot water supply, industrial development, network pressure, network leak, and so on. Factors affecting the reduction of water consumption include water scarcity; the vast introduction of water meters; regulation of pressure in the network; the high cost of water, etc. [3].

The dynamics of water consumption over two 30-year time periods in the last 60 years are completely opposite. From 1960s to 1990s, the water consumption of settlements steadily increased. This was the result of the rapid growth of areas with installed sewage systems, the development of industry, an increase in actual water consumption without proper water accounting, as well as almost ubiquitous leaks in everyday life through faulty water collection devices. In the early 1990s, after briefly remaining at the achieved level, the total water consumption in settlements began to decrease rapidly (by 4–5% per year). The reasons were a sharp drop in industrial production as well as a decrease in specific water consumption on a daily basis [4].

The reduction in water consumption directly affects the disposal of water. According to the report by the Russian authority for statistics, in the whole Russian Federation, the average wastewater consumption in the centralized water supply decreased from 42.3 to 28 million m^3^/day from 1995 to 2013, i.e., by more than 35% [5]. In large cities, the decline even reached 50%. There is no data for a later period, but the trend generally remains. Under seemingly similar conditions of origin, there are no two streams of domestic sewage of the same composition [6]. The differences are caused by:

- reception (or lack of reception) of surface wastewater into the sewage system;
- the level of improvement of the housing stock of settlements;
- the value of specific water consumption;
- the composition of industrial wastewater;
- the intensity of infiltration into pipelines (or exfiltration from pipelines);
- the proportion of concentrated flows, etc.

The performance of treatment facilities for incoming organic pollutants is usually estimated by the population equivalent (PE). In the EU, this characteristic is used for rationing purposes [7].

An important regularity in the formation of the load on the wastewater treatment plants (WWTP) is associated with the concept of PE: a decrease in wastewater consumption caused only by water saving does not lead to a decrease in the load of pollutants on the WWTP but only to an increase in the concentration of these pollutants. During the 20–25-year period of the decrease in the inflow to the WWTP, the real reduction in the pollution load occurred only due to the closure of industrial enterprises in settlements [8].

As in recent years elsewhere, the main direction of improving the WWTP is currently not in an extensive plane (improving cleaning quality and expanding the list of guaranteed pollutants to be removed), but in an intensive plane, and primarily consists of the introduction of energy and resource efficiency technologies. There is a dramatic change in the paradigm of the WWTP with the transition from the task of purification (destruction of pollution) to the task of recovery of all components of wastewater while maintaining the efficiency of purification [2,4].

First, this approach is aimed at the energy sector, at using substances extracted from wastewater to produce energy. For a number of reasons, the main one of which is the objective lack of incentives for the development of alternative energy in the Russian Federation, as well as the abundance of unresolved tasks in terms of wastewater treatment, this direction of modernization of the WWTP in Russia is practically not developing [3,7,8].

Thus, modern engineering science and practice distinguish the following tasks solved by the urban wastewater treatment sub-sector, provided with specially developed technologies and calculation methods:

- removal of coarse impurities, mineral and floating substances;
- removal of organic pollutants (without isolation of individual substances);
- removal of nitrogen and phosphorus compounds;
- disinfection of treated waters;
- treatment of sewage sludge in order to obtain by-products (biogas, organic fertilizer, soil, etc.) or practically non-hazardous or low-hazard waste intended for placement in the environment.

The wastewater treatment sub-sector in the former USSR countries, despite the extremely low availability of specialized equipment, occupied one of the leading places in the world according to the results of its work. Almost all cities were equipped with biological treatment facilities (Table 1). Treatment tasks had to be solved with minimal equipment and capital investment thanks to the advancement of domestic specialists [1,2].

For a considerable period, the development of the sub-sector was directed towards the removal of suspended solids. Considerable funds were invested in wastewater treatment facilities created based on granular filters. The post-treatment stage made it possible to increase the efficiency of wastewater treatment from suspended solids and organic compounds (BOD) by only a few percent. However, the cost of facilities and their operation was up to half the cost of the main purification process. Most of the post-treatment facilities built during that period are currently not working or are operating inefficiently [9].

**Table 1:** Age of existing WWTPs [10].

Year of WWTP Construction	WWTPs According to Their Capacity [%]		
(Age)	>300 k m^3^/Day	100–300 k m^3^/Day	<100 k m^3^/Day
Before 1970 (>50 years)	22.7	20	13
1970–1980 (40–50 years)	50	38	36
1980–1990 (30–40 years)	13.7	25	32
1990–2000 (20–30 years)	4.5	4	13
Since 2000 (<20 years)	9.1	13	6
Were reconstructed since 2010 [%]	57	15	37

In all three performance ranges, the main capacities were built between 1970 and 1985. Large and medium-sized WWTPs were completed until 1990. After 2000, only 6% to 13% of the facilities were built.

Until 1990, the completion of structures and the commissioning of additional blocks were mainly carried out. After 2000, the construction of new blocks and stations was carried out using new technologies with the removal of nitrogen or nitrogen and phosphorus. Reconstructions with changes in technology occurred in 2000–2015. Despite the significant proportions of objects that underwent reconstruction in 2 groups out of three, reconstructive measures mainly concerned the rejection of chlorination in favor of UV treatment and the transition to mechanical dewatering. The share of facilities where nitrogen removal technologies (or nitrogen and phosphorus) are implemented does not exceed 10% [11,12].

Thus, the existing facilities at most of the stations under consideration have good potential for the introduction of modern biological purification technologies, which is currently little implemented. However, two important factors should be taken into account:

- The majority of WWTP were built in the 70 s and 80 s, and during reconstruction, a greater or lesser amount of restoration of the condition of reinforced concrete structures is required. This increases the cost of work from 50% to 500% regarding the technological modernization of the biological purification process (technology changes with equipment replacement) [13];
- The mass load on the WWTP is formed in proportion to the number of residents, and taking into account the discharges of industrial enterprises, the amount of underloading according to design indicators is absolutely not identical to underloading for pollutants. On the WWTP, only mechanical cleaning and settling structures are calculated by consumption; the rest of the structures are calculated by mass load.

As Table 2 shows, the existing treatment facilities may, for the most part, have a certain reserve of tank volumes, which is caused by a smaller amount of incoming wastewater compared to design solutions. This applies to all stages of cleaning and is especially relevant for facilities designed to remove organic pollutants and nutrients. During the reconstruction of sewage treatment plants, the reserve that has arisen with a decrease in wastewater intake can be used to implement modern pollution removal schemes that require, as is correct, more HRT. Furthermore, the available stock can ensure that the stages of modernization are completed without disrupting operations [14,15].

Nevertheless, as already mentioned earlier, a decrease in the hydraulic load can lead to an increase in the amount of pollution in the incoming wastewater. Thus, the volume reserve requires a calculation justification, which has become the subject of consideration in this article.

## 2. Materials and Methods

**Figure 1:**
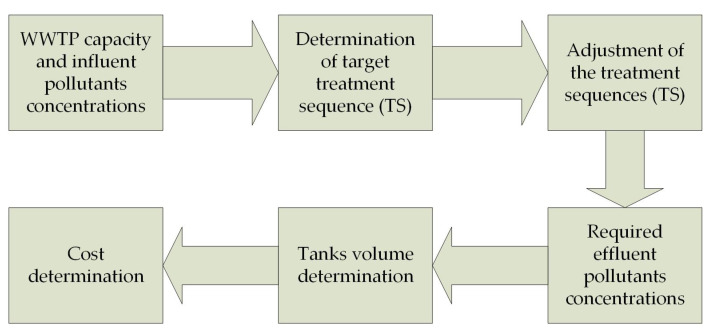
Research sequence.

In the study, an attempt was made to perform a dynamic preliminary project analysis of technological and operational indicators of WWTPs for various solutions of their modernization. Capdetworks 4.0 software (Hydromantis Environmental Software Solutions, Inc.: Hamilton, ON, Canada) was used as a modeling and calculation tool [16,17]. Based on the influent characteristics, the software creates each unit process in a specified process layout, then calculates the cost of the design. Two-step process allowed inspecting the generated design and, if necessary, modifying it using the program’s design override tools. To provide usable calculated designs and make the program simpler to use for planners who need planning-level cost estimates of a new facility or an upgrade to an existing facility, typical design defaults have been utilized for each unit process [18]. The calculation procedure was constructed as follows (Figure 1).

Before the calculation, the initial operational indicators were determined, which were the capacity and concentrations of the main pollutants in the wastewater influent. Then the technological cycle of the WWTP was compiled with the determination of the necessary characteristics of individual processes. The result of modeling and calculation was the determination of output parameters—the concentration of pollutants in the effluent, the volume of tanks and the areas occupied by them, and energy consumption for various technological needs. The necessary adjustments were carried out in order to obtain results that were close to the operating parameters of the existing treatment facilities. The calculation of the tank volumes was carried out based on the required hydraulic retention time (HRT). The calculation of energy costs was carried out on the basis of the technological needs of the purification processes and the required equipment capacity.

In order to investigate the impact of individual operational indicators on the cost indicators of the entire WWTP, several values of WWTP capacity were considered within the framework of the study: 1000, 5000, 10,000, 20,000, 50,000, and 100,000 m^3^/day. The selected values correspond to the approximate population range of 5 to 400 thousand people, which includes most of the settlements in the Russian Federation.

As was noted earlier, a significant part of the treatment facilities has already been erected, so the task of their modernization or reconstruction is the most relevant and interesting.

In the course of the study, three technological schemes with a classical sequence of treatment processes, which were implemented at most existing facilities, were considered: a preliminary treatment, primary treatment, secondary treatment, and disinfection. A common scheme with sludge thickeners for waste activated sludge (WAS), anaerobic digestion of a mixture of WAS and primary sludge, and its subsequent dewatering has also been adopted as a sludge treatment facility.

**Figure 2:**
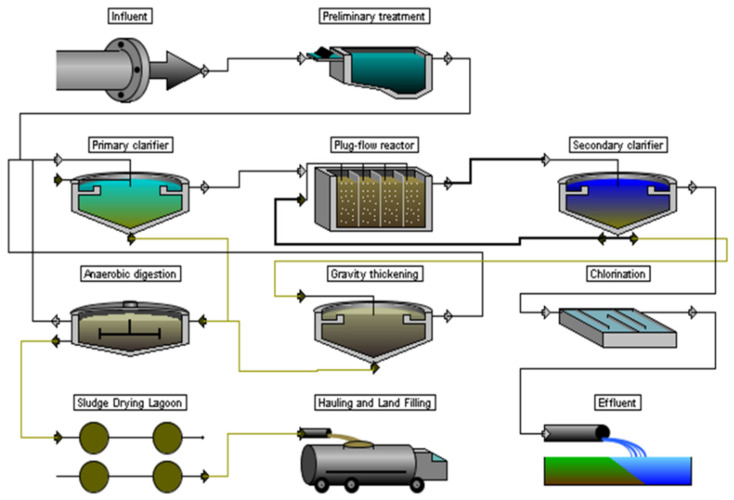
Treatment sequence 1.

The first treatment sequence (TS1) can be called conventional since it was quite common until the 1990s (Figure 2). Many existing treatment facilities used this scheme or its closest analogues.

A plug-flow activated sludge reactor (ASR) in combination with a secondary clarifier (SC) was used as the secondary treatment stage. Chlorination was used for disinfection. Sludge dewatering occurs in natural conditions on sludge drying lagoons. When using such a treatment scheme, the most complete removal of only organic (or carbon) pollution (BOD/COD indicators) is possible, while the removal of nitrogen (N) and phosphorus (P) does not exceed 5% and 1%, respectively.

During the research, it was noted that in the Capdetworks software package TS1 can be analyzed for two sub-modes of operation. The first sub-mode (TS1a) is carbon removal only—the so-called complete biological purification according to the terminology common in the Russian Federation and neighboring countries. This mode is characterized by the removal of impurities by BOD and suspended solids to values of about 5–15 mg/L. The removal efficiency of nitrogen and phosphorus compounds is rarely greater than 5–10%, and the BOD ratio is used for calculations BOD:N:P = 100:5:1.That ratio means, that for every 100 mg/L decrease in the concentration of pollutants by BOD, there is a decrease of 5 mg/L and 1 mg/L for nitrogen and phosphorus, respectively. In Russia and neighboring countries, similar technological solutions were typical at WWTPs until the early 2000s, when the requirements for the quality of treated wastewater were tightened.

The second sub-mode (TS1b) implies not only the removal of organic pollutants, but also the partial removal of nitrogen—nitrification, in which the oxidation of ammonium to nitrites and nitrates occurs without their subsequent reduction. As a rule, this mode of operation requires a higher value of HRT, so it is often called “extended aeration”. This sub-mode can be called the simplest way to modernize (reconstruct) the WWTP of the first sub-mode. Most often, it was used in the case of a decrease in the amount of influent, so the HRT in the reactor increased “naturally”. Taking into account the real state of affairs in the water sector (Table 2), when a decrease in wastewater occurs by 60–80% of WWTP, the transition to such a treatment mode would not require significant costs.

Due to the above, the WWTPs with similar treatment sequences require reconstruction, and for current research it will be a sort of control treatment sequence. The second and third treatment sequences (TS2 and TS3) will, in turn, be possible solutions for the modernization of TS1.

If the key indicators (TSS, BOD, nutrients (N and P)) are considered, then the majority of the pollutants are removed at the secondary treatment stage, which is, in fact, a key element of any technological scheme for wastewater treatment. This stage of purification is the most energy-intensive. Thus, within the framework of the research, the main attention was paid to comparing the costs of the biological purification stage for various treatment sequences. In addition, the modernization of the stage of biological purification is the most difficult; therefore, the justification of the application of certain solutions becomes especially important [19].

**Figure 3:**
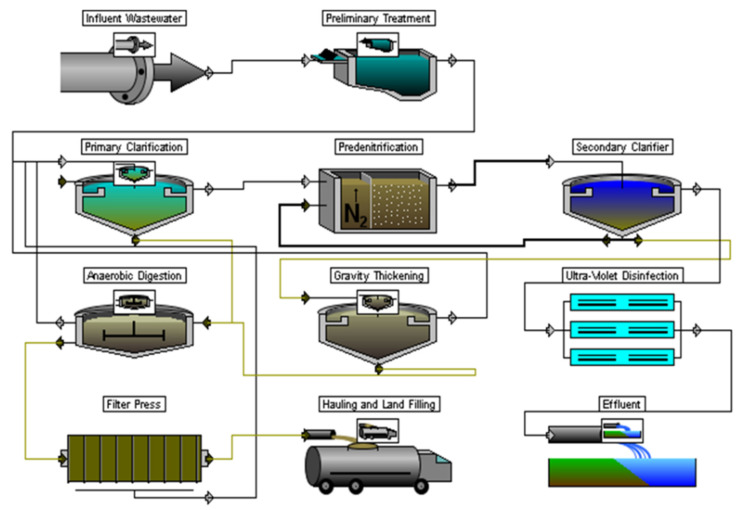
Treatment sequence 2.

For the TS2, the scheme of secondary treatment with ASR for N/P removal and SC was adopted (Figure 3).

**Figure 4:**
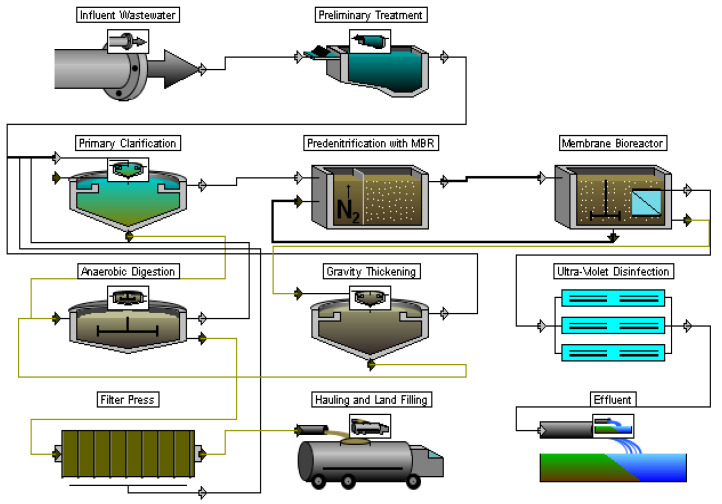
Treatment sequence 3.

For the TS3, a technology with a membrane bioreactor (MBR) was adopted, in which the ASR is equipped with submersible membrane modules. The separation of sludge and wastewater here is carried out by a membrane method (Figure 4); therefore, a SC is not required. As a result, the application of MBR allows achieving comparable results at one stage of the purification process, as with the use of ASR for N/P removal with tertiary treatment, the increased MLSS, which requires an increase of its area by 2–4 times, and the volume of SCs by at least 2 times. Thus, application of MBR affords reduction of the area of the entire secondary treatment stage by 2–3 times and the volume by 3–4 times compared with the use of the SCs.

Unlike TS1, more effective UV-treatment methods were used at the disinfection stage for TS2 and TS3, and dewatering was done on filter presses (rather than sludge-drying lagoons). Thus, the composition (not the number of individual facilities, their dimensions, etc.) of the preliminary and primary treatment, disinfection facilities, and sludge treatment for TS2 and TS3 was the same; the differences were only at the stage of secondary treatment. At the same time, it should be noted that the design of secondary treatment (depending on the technology used) may affect the design and technological features of other facilities.

The operation of the ASR + SC combination in Russia has been well studied both from a technological and economic point of view. The combination of ASR with MBR remains rare in practice; therefore, the calculated indicators obtained in this study may have some practical significance for further substantiation of the use of this technology. Thus, within the framework of the study, four modes of operation of technological cleaning schemes were considered.

## 3. Results

As initial data, the concentrations of pollutants were calculated by the main indicators based on the specific amount of pollutants coming per capita (Table 3). This indicator is used, in particular, in calculations when reliable actual data on wastewater are not available and only the population is known. The calculation was carried out using Equation (1). The initial values generally correspond to the average quality of municipal wastewater entering the treatment facilities.

(1) $$C_{i}=\frac{1000\cdot A_{PC}}{Q_{PC}}$$

where *A_PP_* is the daily amount of pollutants per capita;

**Table 3:** Specific values of pollutants amount per capita and initial pollutants concentrations [20].

Indicator	Amount of Pollutants per Capita, *A_PC_* [g/Day]	Pollutants Concentration [mg/L]
Total suspended solids (TSS)	67	250
Biochemical oxygen demand (BOD_5_) of untreated water	60	220
Chemical oxygen demand (COD)	120	440
Ammonia (NH_4_)	8.8	33
Phosphates (PO_4_)	1.0	3.7

*Q_PC_* is the average daily wastewater production (L per capita day^−1^); for further calculation, it was accepted as 270 L per capita day^−1^.

Before starting the calculation, the main operation characteristics for each TS were determined. For conventional schemes with gravity separation of suspended solids (TS1a and TS1b), MLSS values may be applied at the conventional level (about 3 g/L) with the sludge retention time (SRT) value of 6 days. For TS2 or similar sequences, the MLSS value can be higher (4–5 g/L), which is quite common to achieve a higher quality of treatment or in the case of increased concentrations of pollutants in the influent wastewater. It is important to note that increasing the MLSS in conventional ASR can be difficult since this inevitably leads to an increase in the load on the SC, an increase in the amount of WAS, and the cost of its processing, respectively. SRT for TS2 was 15 days.

The MLSS value applied for the operation of the MBR is usually in the range of 6 to 12 g/L [14]. For the simulation under study, an average value of 10 g/L was adopted [13]. In addition, special technological parameters have been adopted for TS3, which relate exclusively to the use of membrane cleaning technologies: membrane flux (20 LMH) and membrane density (450 m^2^/m^3^). The membrane packing density corresponds to Kubota hollow-fiber membrane cassettes [21]. SRT for TS3 was 15 days.

Table 4 shows the average values of contamination concentrations in discharged wastewater calculated using the Capdetworks 4.0 software package after appropriate calibration. It is an important condition for calculating the convergent indicators with the indicators of real wastewater treatment plants. Therefore, the adjustment of operating parameters for each treatment sequence was carried out iteratively. As a result, the values that can be achieved using these secondary treatment facilities were obtained.

**Table 4:** Pollution values applied in the calculation.

Indicators	Influent	Effluent				Limit Value [22,23]
		TS1a	TS1b	TS2	TS3	
BOD_5_ [mgO_2_/L]	220	4.75	3.45	2.3	1.6	2.1
COD [mgO_2_/L]	440	15	13.1	12.5	5.4	15
TSS [mg/L]	250	10	10	10	3.0	BP * + 0.25
N-NH_4_ [mg/L]	33	24.4	1.2	0.38	0.4	0.4
N-NO_2_ [mg/L]	-			0.05	0.02	0.02
N-NO_3_ [mg/L]	-		28.5	10	9.0	9.0
P-PO_4_ [mg/L]	3.7	3.57	3.5	0.4	0.02	0.2

Note: BP *—background pollution in the river before wastewater discharge.

Table 4 demonstrates that the calculated values for TS2 and TS3 generally correspond to the current regulatory requirements; that is, technologically, both sequences can help to achieve required values of pollutant concentrations. It was not possible to achieve the desired efficiency for TS1a or TS1b, as expected. After the calculated values were achieved, it was revealed that Capdetworks software allows obtaining relatively approximate values of pollutants’ indicators.

**Figure 5:**
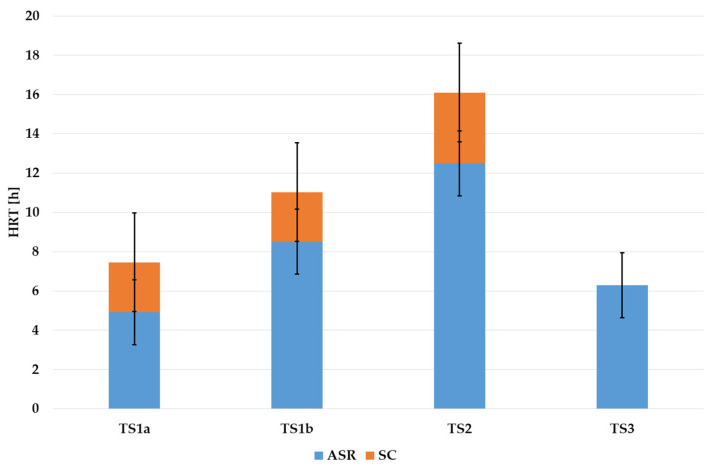
Values of hydraulic retention time (ASR and SC) for treatment sequences under research.

This approach demonstrates a sort of overall view, but the precision of the calculation may not be high enough. For instance, HRT values remained approximately constant in the case of the same treatment sequence but for different capacities. In real WWTP, this condition may not always be fulfilled. Figure 5 shows mean values of HRT for each treatment sequence for ASR and SC. When comparing HRT in ASR, it is clear that higher HRT values are required for higher treatment efficiency: TS1a provides lower treatment efficiency than TS1b, TS2, and TS3. TS3, however, may be considered an exception due to special conditions of operation of this treatment process (MBR) due to the higher value of MLSS in the activated sludge reactor. If to compare the overall values of HRT for the ASR + SC system, TS3 shows the smallest volume among studied treatment sequences as TS3 requires no secondary clarifiers. TS1a and TS1b both require SC, which volume should provide HRT = 2.5 h. TS2 require SC volume for HRT > 3 h.

**Figure 6:**
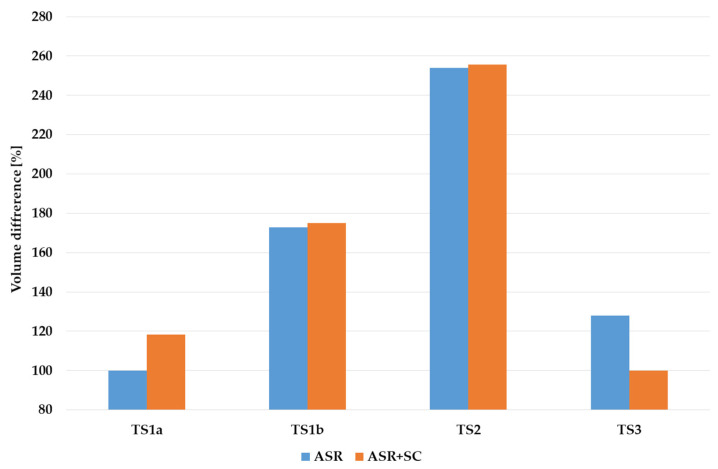
Difference in values of hydraulic retention time.

Figure 6 gives a sort of summary for Figure 5 and Figure 6. If minimum HRT (among treatment sequences) is set equal to 100%, the required extension may be seen. TS1a require minimum HRT in ASR, and TS3 require approximately 25% higher HRT. If to consider both ASR and SC, than TS1a requires HRT, which is 18% higher than for TS3. TS1b and TS2 require higher HRT with the factor of 1.8 and 2.4, correspondingly, related to minimum values.

To summarize, the difference between HRT for various TS naturally results in an appropriate difference in the volume of tanks needed for treatment. Moreover, different volumes require different capital costs, though the difference is unlikely to be linear. Lower/higher HRT also has an effect on the area needed for placement of the facilities, which is crucial in the case of WWTP modernization when the area is limited and, in most cases, cannot be extended. Alternatively, application of TS3 or other schemes with membrane bioreactors may be especially efficient in cases when the capacity should be extended under limited available volume and/or area [24].

It should be noted that the real treatment efficiency is not a constant value due to the possible fluctuations in the influent wastewater composition. In the case of such conditions, the operating experience of membrane bioreactors demonstrates a low dependence of the cleaning quality on sharp fluctuations in the composition of contaminants, which indicates a higher stability of the system compared to other technological solutions [17,18].

Speaking about the reduction in the need for construction volumes (as one of the key components of capital costs) in the case of using MBR, it should be mentioned that another significant expense item has arisen, which ensures a reduction in volumes—in fact, the purchase of membrane modules [21,25]. At the same time, the costs of their acquisition can be conditionally attributed both to capital, that is, carried out at the construction stage, and to operational, since due to the service life of the membranes (7–10 years) during operation, there will be a need for their periodic replacement. In the following, the approximate cost of membranes required for a membrane bioreactor at facilities with a daily capacity of *Q_daily_* = 1000 m^3^/day will be made:

(2) $$C_{membr}=\frac{Q_{des}\cdot 1000}{J}\cdot C_{1}\;\left[EUR\right]$$

where *Q_des_* is the design (maximum) flow [m^3^ h^−1^]:

(3) $$Q_{des}=\frac{Q_{daily}}{24}\cdot K_{gen.max}\;\left[m^{3}h^{-1}\right]$$

*K_gen.max_* is the coefficient of irregularity of the wastewater flow (see Table 5).

For *Q_daily_* = 1000 m^3^ day^−1^. *K_gen.max_* is equal to 2.06. Then:

Qdes=100024·2.06=86m3h

*C*_1_—Kubota membrane costs taken equal to 48 *EUR* per 1 sq.m [26];

*J*—permeate flux that was taken equal to 20 L m^−2^ h^−1^.

Using Equation (2) with constant values of *C*_1_ и *J* and variable value of *Q_des_* (considering *K_gen.max_*) approximate membrane costs can be calculated for WWTP with capacity in the investigated range.

$$C_{membr}=\frac{86\cdot 1000}{20}\cdot 48=206,400\;EUR$$

The results of cost calculations are presented in Table 6, which also shows the values of the specific annual membrane costs attributed to 1 cubic meter of the daily capacity of the WWTP, taking into account the service life of the membranes is equal to 10 years.

Table 6 reveals that the calculated cost values are considered significant, but the share of the cost of membranes in the total costs will decrease as capacity increases. Moreover, since 2010, a tendency has been witnessed to a reduction in the cost of the membrane modules due to the growing number of their manufacturers, which may in the future contribute to their wider use. It also should be noted that application of the membrane requires regular chemical cleaning, normally by means of citric acid. However, the cost of chemical cleaning did not exceed 5% of specific membrane costs in *EUR* m^−3^ year^−1^.

In addition, during the calculation, the electricity consumption due to the operation of the overall treatment facilities and secondary treatment block in particular were considered (Table 7). The energy consumption for the treatment plant was obtained in an enlarged manner, however, for secondary treatment facilities, itemization was carried out according to technological needs. So, for TS1a and TS1b, the costs of aeration were taken into account, as well as for the operation of a SC, the recycling of return activated sludge (RAS) and the removal of waste activated sludge (WAS). For TS2, the costs of aeration and internal recycle of the sludge mixture were considered, as well as the operation of a SC, the recycling of return activated sludge (RAS) and the removal of waste activated sludge (WAS). For the MBR (TS3) scheme, the costs of aeration, recycling of RAS and removal of WAS were estimated, to which energy costs were added for pumping out the entire flow of purified water by vacuum pumps, as well as the costs for MBR operation, which consists of air supply for blowing membranes and physical cleaning by means of backwashing. It is the last point that provides a significant increase in electricity costs when using MBR. Additionally, for each of the treatment sequences, the specific consumption of electricity per 1 cubic meter of plant capacity is calculated.

**Figure 7:**
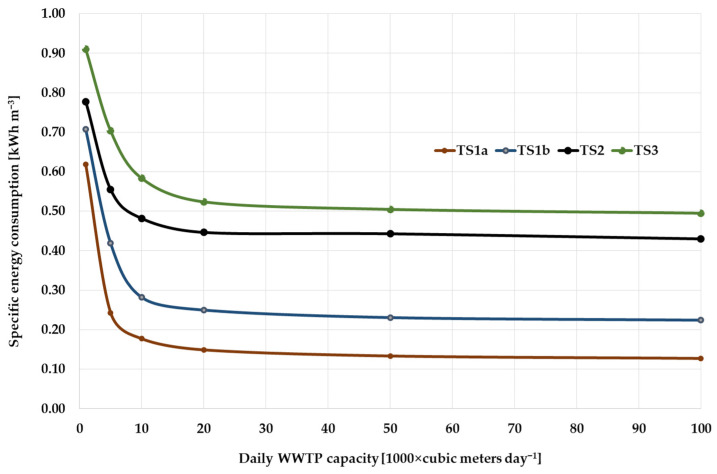
Specific energy consumption.

When analyzing the boundary values of the WWTP capacity considered in the article—1 and 100 thousand m^3^/day—the values of the specific electricity consumption for them differ significantly from all four TS. The greatest differences are observed in the cases of TS1a (almost 5 times) and TS1b (3 times). This can be explained by the fact that, for small-capacity WWTPs, energy-intensive equipment has a significant impact on overall energy consumption, and as productivity increases, this influence will be mitigated. In addition, the features of the Capdetworks software package that were revealed during work with it can explain such results. The libraries of the software package contain a limited set of equipment used in calculations, and this equipment may have overestimated (compared to the necessary) characteristics for small-capacity WWTPs. If we look at the graphical interpretation (Figure 7), we can see that for TS1a and TS1b there is a significant decrease in specific energy consumption with an increase in consumption from 1000 to 5000 cubic meters per day. At the same time, for TS2 and TS3 (in which more energy-intensive equipment is used), the differences in the specific consumption of electricity for the boundary values of the consumption are 1.5–2 times.

**Figure 8:**
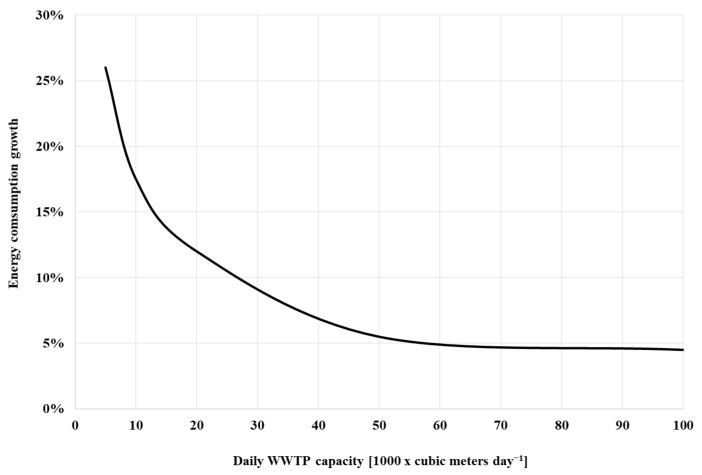
Difference in specific energy consumption for TS2 and TS3.

Analysis of the results in Table 7 and Figure 8 shows that when the flow rate changes from 1 to 20 thousand cubic meters per day, the most intense decrease in specific energy consumption is observed. With a further increase in production to 50 and 100 thousand cubic meters, the specific energy consumption decreases in the range of 10–15%. In general, it can be noted that the characteristics of energy consumption are similar for all cleaning sequences. The obtained calculations showed that the differences between the simplest of the considered technological schemes (TS1a) and the most advanced (TS3) in terms of energy consumption are 3.5 times, which generally corresponds to previously conducted studies. However, this comparison cannot be called correct since the quality of treatment in both cases is significantly different. From a practical point of view, the most interesting comparison is between TS2 and TS3, which are able to provide comparable cleaning quality. If to consider the differences in the values of specific electricity costs, then in the entire range of the study, this indicator is greater for TS3, while at a capacity of 1000 m^3^/day the difference is 26%, and further decreases to 4.5% at a capacity of 100,000 m^3^/day (Figure 8). In general, the data obtained during modeling are similar to the previously described data [27,28], but they, like all model indicators, require validation on real objects.

Thus, comparing the results obtained only for TS2 and TS3, it can be concluded that as capacity (flow rate) increases, the difference between these two treatment sequences in energy consumption decreases, while TS2 still requires significantly larger areas and volumes of structures due to the higher value of HRT. In the case of new construction, this suggests that the use of the TS3 will make the structures more compact. In the case of reconstruction, with proper justification, this will ensure greater capacity in the existing volumes of structures [29,30].

## 4. Conclusions

Based on the results of the calculations carried out (including using the Capdetworks 4.0 software package), the following conclusions can be drawn:

1. During the calculations, it was discovered that using MBR for sludge separation reduces the required volumes of capacitive structures by approximately 60%. This may also result in significant reduction of capital costs for WWTP construction.
2. It is determined that the cost of purchasing and updating membranes is a significant expense item, and the specific costs (for the purchase of the considered type of membranes without additional equipment) may reach 15.5–21.5 EUR per cubic meter of treated wastewater. At the same time, the cost of membranes has recently tended to decrease.
3. The use of membranes increases the specific cost of electricity by 10–25% compared to technological schemes that permit achieving a similar quality of water purification.
4. The use of membrane technologies at the stage of sludge separation can have significant potential in the reconstruction of structures in conditions of increased productivity and limited opportunities for expanding the area.

## Figures and Tables

**Table 2:** Distribution of hydraulic load at existing WWTPs [10].

Load Estimate Related to	WWTPs According to Their Capacity [%]		
WWTP Capacity	>300 k m^3^/Day	100–300 k m^3^/Day	<100 k m^3^/Day
Slight overload (105–115%)	0	11	1
Designed load (70–100%)	21	27	12
Significantly under-loaded (50–70%)	48	42	19
Low load (less than 50%)	32	20	68

**Table 5:** Values of the irregularity coefficient [20].

Average Flow [L/s]	5	10	20	50	100	300	500	1000	>5000
*K_gen.max_*	2.5	2.1	1.9	1.7	1.6	1.55	1.5	1.47	1.44

**Table 6:** Membrane costs.

Daily Flow [m^3^/day]	Design Flow	Membrane Costs [1000 × *EUR*]	Chemical Cleaning Costs [1000 × *EUR* Year^−1^]	Specific Costs
	[m^3^/h]			[*EUR* m^−3^ Year^−1^]
1000	86	206.4	0.9	21.54
5000	350	840	4.1	17.6
10,000	667	1601	8.1	16.8
20,000	1308	3139	16.2	16.7
50,000	3125	7500	40.5	15.8
100,000	6125	14,700	81	15.5

**Table 7:** Energy indicators.

Process	Energy Consumption [MWh Year^−1^] with WWTP Capacity [1000 m^3^ Day^−1^]					
	1	5	10	20	50	100
	**TS1a**					
Aeration in ASR	48.1	177	337	674	1690	3400
RAS recycle	3.9	19.3	38.6	76.9	192	383
SC operation	7.5	8.1	9.1	10.20	14.5	21.8
WAS discharge	0.2	0.9	1.6	3.6	9	18
Overall for secondary treatment	59.7	205.3	386.3	764.7	1905.5	3822.8
Overall for WWTP	225.8	443.5	646.8	1087.1	2435.5	4645.2
Specific energy consumption [kWh m^−3^]	0.62	0.24	0.18	0.15	0.13	0.13
	**TS1b**					
Aeration in ASR	76	344	688	1380	3590	6880
RAS recycle	4	19	39	77	191	382
SC operation	8	8	9	10	15	22
WAS discharge	0.2	0.9	1.6	3.6	8.5	16.0
Overall for secondary treatment	88	372	737	1471	3804	7300
Overall for WWTP	258	765	1029	1823	4210	8177
Specific energy consumption [kWh m^−3^]	0.71	0.42	0.28	0.25	0.23	0.22
	**TS2**					
Aeration in ASR	129	594	1210	2380	5940	11,300
Internal recycle	11.4	56.9	114	227	567	1410
RAS recycle	7.4	36.8	73	146	365	728
SC operation	7.5	8.6	9.7	10.2	15	26.5
WAS discharge	0.13	0.7	1.3	2.8	7	12.5
Overall for secondary treatment	155.43	697	1408	2766	6894	13,477
Overall for WWTP	284	1013	1758	3387	8177	16,032
Specific energy consumption [kWh m^−3^]	0.78	0.56	0.48	0.46	0.45	0.44
	**TS3**					
Aeration in ASR	76.7	390	784	1560	3900	8070
Internal recycle	1.7	8.4	16.7	33.2	83.2	165
RAS recycle	44.7	223	252	503	1250	2510
MBR operation	71.9	359	718	1280	2870	5480
Permeate pumping	8.1	40.2	81	161	403	804
WAS discharge	0.1	0.6	1.1	2.3	5.6	11.2
Overall for secondary treatment	203.2	1021.2	1852.8	3539.5	8511.8	17,040
Overall for WWTP	332.3	1284	2129	3823	9210	18,065
Specific energy consumption [kWh m^−3^]	0.91	0.70	0.58	0.52	0.50	0.49

## Data Availability

Not applicable.
